# Addressing the health advocate role in medical education

**DOI:** 10.1186/s12909-020-1938-7

**Published:** 2020-01-30

**Authors:** Suzanne Boroumand, Michael J. Stein, Mohammad Jay, Julia W. Shen, Michael Hirsh, Shafik Dharamsi

**Affiliations:** 1 Faculty of Medicine, University of Ottawa, Ottawa, Ontario Canada; 2 Department of Surgery, University of Ottawa, Ottawa, Ontario Canada; 3 College of Health Sciences, The University of Texas at El Paso, El Paso, TX USA

**Keywords:** Social determinants of health, Interprofessionalism, Advocacy, Medical education

## Abstract

The health advocate role is an essential and underappreciated component of the CanMEDs competency framework. It is tied to the concept of social accountability and its application to medical schools for preparing future physicians who will work to ensure an equitable healthcare system. Student involvement in health advocacy throughout medical school can inspire a long-term commitment to address health disparities. The Social Medicine Network (SMN) provides an online platform for medical trainees to seek opportunities to address health disparities, with the goal of bridging the gap between the social determinants of health and clinical medicine. This online platform provides a list of health advocacy related opportunities for addressing issues that impede health equity, whether through research, community engagement, or clinical care.

First implemented at the University of British Columbia, the SMN has since expanded to other medical schools across Canada. At the University of Ottawa, the SMN is being used to augment didactic teachings of health advocacy and social accountability. This article reports on the development and application of the SMN as a resource for medical trainees seeking meaningful and actionable opportunities to enact their role as health advocates.

## Background

The Health Advocate role is a vital pillar of the CanMEDs competency framework, requiring future physicians to develop key capabilities that would enable them to address the social determinants of health and improve health outcomes at the level of the patient, the community, and the populations served. However, it has received an underwhelming response by Canadian medical educators, physicians, and students [1]. The effective integration of health advocacy related competencies into medical education remains a challenge [2–7]. Canadian physicians surveyed in 2012 indicated that health advocacy is perceived to be of lesser significance among the CanMEDs roles [8]. Similar findings were observed in European studies [9, 10]. Some scholars believe that the lack of socioeconomic diversity in the medical school class demographic profile is partly to blame [6]. Others point out that medical education offers limited opportunities to work with socially disadvantaged and marginalized segments of the population, which is required to instrument the knowledge and skills necessary to be an effective health advocate [1, 6, 9, 11, 12]. Medical programs that do provide experiential and community service learning initiatives with a focus on health advocacy and the social determinants of health reported that these types of authentic learning activities are key to enabling students to gain a greater insight into how health advocacy can be applied [13, 14]. Students are enabled to develop a greater appreciation of the root causes and subsequent effects of health disparities, and a greater sensitivity to the circumstances of people in society who are rendered vulnerable because of various socioeconomic factors that prevent them from protecting and preserving their well-being [15–19]. In response to the deficit, the Committee on the Accreditation of Canadian Medical Schools (CACMS) and the Liaison Committee on Medical Education (LCME) in the United States officially adopted a new accreditation standard requiring medical schools in North America to include service-learning opportunities to medical students to address this gap.

At the health system-level, physicians are expected to work alongside communities to advocate for change that reflects principles of social accountability [11]. However, many physicians feel frustrated when faced with patients from complex socioeconomic backgrounds, expressing reluctance to engage in discussions around patients’ social situations [9, 12]. The low emphasis on health advocacy is likely multifactorial. For one, the health advocate role has been noted as difficult to teach and assess [1]. In addition, medical school admissions committees have habitually placed a higher value on students’ academic scores, with little attention to their experience and understanding of social issues [15]. Furthermore, time constraints are a likely contributor to physicians avoiding discussions around the social causes leading to poor health outcomes [20]. Finally, the majority of medical training occurs in academic institutions, distant from community areas where some marginalized populations live [21].

To address these challenges, in 2013, a small group of medical students and residents at the University of British Columbia, with faculty support, established an online platform to assist medical trainees to seek out meaningful opportunities to enact the CanMEDS role of the health advocate [22]. The aim of this platform, named the *Social Medicine Network* (SMN), was to provide an online resource that would help students identify health providers and/or agencies whose work would enable them to bridge the learning gap between *what they are taught* about the social determinants of health and *what to do* to address the related health issues that subsequently arise. The online platform was subsequently adopted and adapted by the medical students at the University of Ottawa, and endorsed by the Canadian Federation of Medical Students. In this paper, we report on the development and components of SMN, while linking its role to experiential learning of advocacy.

### The Social Medicine Network (SMN)

The Social Medicine Network is a web-based resource that lists clinical, service, and research related opportunities where medical trainees can gain practical knowledge and skills to address the social determinants of health and respond to health disparities that vulnerable populations may face [22]. Trainees are able to find and select clinical, community or research related opportunities that align with their own interests and commitments toward addressing issues that impede health equity. Opportunities are available in a wide range of areas: Addictions Health, Environmental Health, Health Education and Advocacy, Immigrant and Refugee Health, Indigenous Health, LGBTQ Health, Mental Health, Prison Health, Homelessness, and Women’s Health. The website is maintained by a group of students who have both the technical ability to maintain it, and the commitment and inspiration to find and recruit organizations using templates listed in Additional file 1, that can provide the relevant clinical, service, and action research opportunities. Ideally, the opportunities ought to help trainees develop the skills necessary to becoming an effective health advocate. Once the organization has consented to providing students with a volunteer, research, or clinical placement opportunity, the contact information including the address, phone number, email address, and description of the opportunity and role provided by the organization is included on the website under each category. Medical trainees are then encouraged to contact the organizations by email or phone for opportunities they are interested in, and subsequently liaise with the organization’s administrator to arrange for a start date. The organizations will then share with the trainees the protocols and actions necessary to get started with the process.

#### Community engagement is key

Effective health advocacy is perhaps more crucial today than ever before [23]. Traditional didactic methods of teaching are insufficient in fostering passion and skills for health advocacy. Akin to teaching clinical knowledge through bedside teaching, an effective way of teaching health advocacy is through community involvement that actively involves learners in the advocacy process [11]. According to a systematic review that analyzed 57 peer-reviewed articles on service learning or community-based education in US medical schools, educators found it easier to use community learning to teach complicated or abstract topics such as cultural competence, the social determinants of health, and professionalism, compared to traditional didactic methods [24–34]. Students who engaged in authentic, hands-on activities developed deeper insights and understanding of related issues and how to better address them [24–34]. Community engagement allows learners to observe and work to address health disparities first-hand, enhancing their understanding of the practical relevance of health advocacy, and fueling their passion for finding solutions to what may appear to be insurmountable issues [3, 11, 16, 17, 35–40].

In a recent case study by the University of British Columbia (UBC), medical students investigated the impact of a community-based learning opportunity on their sense of appreciation around the importance of social accountability and health advocacy [13]. The opportunity provided second-year medical students the ability to take part in a community-service option as an alternative to classroom-based learning, providing medical students the opportunity to work directly with vulnerable and marginalized segments of the population. Students who were interviewed following their experience with the program reported they developed a deeper insight into the disparities that vulnerable populations experience. They learned how marginalization leads to exclusion from meaningful participation in society. Marginalized segments of the population face persistent hardships resulting from discrimination, social stigma, and stereotypes. Armed with a deeper understanding of the lived experience of marginalization, the students learned to establish positive relationships with vulnerable segments of a population and became inspired to engage in sustained efforts to effect positive change through meaningful involvement. The insight the students gained helped to nurture their passion to become actively involved in health advocacy throughout their careers.

#### Integrating the social medicine network in medical education

While social accountability and health advocacy are addressed in medical curricula, teaching is largely didactic in nature and disconnected from the lived experiences of vulnerable populations [16, 41]. In response, the Social Medicine Network Ottawa (SMNO) developed a web-based portal that is simple, convenient, and self-directed, allowing students to access the website whenever necessary, and organize participation in opportunities for trainees based on mutually convenient schedules and interests [2]. Being that this is a volunteer-based portal, which is not associated with any curricular credit, the initiative students take is completely by interest and a genuine commitment to address health disparities. Interested students are provided with the website URL to access the portal. This ensures students are not forced to get involved in initiatives they are not experienced with or passionate about, but instead the platform provides the resources necessary to inspire social responsibility and health advocacy.

Additionally, the nature of the platform allows a multidisciplinary approach and collaboration on issues related to the social determinants of health, which can be accessed on the discussion board. This allows healthcare professionals from different domains (i.e. nurses, physiotherapists, occupational therapists, social workers, et cetera) to stimulate deliberation, brainstorm solutions, and create health advocacy initiatives using an interprofessional approach.

At the University of Ottawa, medical students learn about social accountability and health advocacy through lectures and panels hosted by healthcare providers and community organizers as well as through a mandatory 30 h community service learning placement. Social accountability is taught through the SIM course.

Society, the Individual and Medicine [42]. The course covers a wide curriculum taught using multiple pedagogies including small group teaching, patient interviews, and self-learning modules [43]. The course faculty are appointed on the Social Accountability Leadership Committee and report to the director of Social Accountability [43]. As part of a Social Accountability framework, a student-run Social Accountability Student Advisory Committee (SASAC) was also created to give students who are passionate about addressing health inequities an active voice both independently and in collaboration with the Social Accountability Leadership Committee. SASAC students have been involved in different leadership committees, advocating for innovative, hands-on, and community-based health advocacy curriculum.

As a group, the SASAC find that experiential learning, and fostering connections between community organizations, healthcare providers, and healthcare trainees, are critical for operationalizing health advocacy, a concept which was the motivation behind implementing the SMN model [3]. SASAC members are responsible for updating the SMNO website on community-based health advocacy opportunities and promoting the platform to medical students, residents, and undergraduate students interested in health equity. Medical school faculty ensure that the directive is handed over to new SASAC members annually, ensuring initiative sustainability. The faculty supervisor also provides mentorship for students involved in the opportunities offered through SMNO and provides support in instances where students face challenges along the way. Unlike the mandatory community service learning component of the curriculum, medical students are not given credit on their medical student performance record for participation in an SMNO promoted opportunity. However, they are able to include this work on their resume. The aim of the SMNO initiative is to cultivate an intrinsic motivation to address health inequities, and to provide practical learning opportunities to those who seek it because of a deep commitment to health advocacy.

### Limitations

To date, the positive impact of this initiative is based on self-reported data. A comprehensive impact assessment is needed to ascertain the effectiveness of the SMN in helping medical trainees become successful health advocates.

## Conclusion

The Social Medicine Network was founded on the principle that social accountability is essential for anyone working in healthcare. It brings together an interdisciplinary group of healthcare professionals, trainees, and community-based organizations to work together and enhance an understanding of the broad range of social factors affecting health and well-being. This article is written to highlight the innovative method of educating medical trainees using the Social Medicine Network for the often neglected CanMEDS role, the health advocate. This framework supplements the already existing didactic curriculum that exists in most medical schools, supplementing it with a more experiential-based learning modality. By creating a Social Medicine Network at every medical training institution, we will be working toward addressing the gaps in developing physicians who are feeling more competent as healthcare advocates.

The “Global Consensus for Social Accountability of Medical Schools”, urges ongoing efforts among medical schools to identify increasingly innovative ways of teaching social accountability [44]. Teaching and assessing health advocacy, an essential CanMEDS competency, has proved to be a challenging feat. Nevertheless, the SMN has been successful in engaging trainees to address the social determinants of health and to promote social accountability in practical and sustainable ways. The model has generated interest at other universities in Quebec, Ontario, and Nova Scotia, where SMN chapters will be similarly established.

## Additional file

**Additional file 1.** These templates illustrate the pre-set emails students on the Social Accountability Student Advisory Committee used to recruit volunteer organizations, physicians, shelters, and research departments to be provide opportunities that could be posted on the Social Medicine Network for students to choose from. The blanks were filled in with information that was relevant to the receiving volunteer organization, physician, shelter, or research department.

## Data Availability

Not applicable.

## Abbreviations

LGBTQ: Lesbians, gays, bisexuals, transgenders, and queer
SASAC: Social Accountability Student Advisory Committee
SMN: Social Medicine Network
SMNO: Social Medicine Network Ottawa
UBC: University of British Columbia
WHO: World Health Organization
