# Supplementary material for: Addressing the health advocate role in medical education
Source: BMC Med Educ. 2020 Jan 30;20:28. doi: 10.1186/s12909-020-1938-7 (PMC6993364; doi:10.1186/s12909-020-1938-7)
Supplement: Supplementary file 1 — Additional file 1. These templates illustrate the pre-set emails students on the Social Accountability Student Advisory Committee used to recruit volunteer organizations, physicians, shelters, and research departments to be provide opportunities that could be posted on the Social Medicine Network for students to choose from. The blanks were filled in with information that was relevant to the receiving volunteer organization, physician, shelter, or research department. [file 12909_2020_1938_MOESM1_ESM.docx]

**Appendix A**

Good morning (afternoon)

My name is ____, and I am a medical student at the University of Ottawa.

I am part of a team of medical students that are putting together a Social Medicine Network - an online resource of social or community health services offering community contacts. This is a student-run project to create a website for other students to be able to visit and find learning opportunities in various topics related to social accountability. We would be honoured to list your [service/organization/initiative].

I am writing to you because we are interested in listing (x opportunity or contact) as a resource in the Network. This would make this opportunity (or contact), along with others, more visible to students who are interested in this work and/or learning more.

Please let me know if (contact x or service x) is interested in being included in this initiative. If so, we will ask for some basic information in order to complete the listing on the Network.

Ultimately, we hope the Social Medicine Network will help bridge the gap between social and clinical medicine, allowing students to appreciate the interconnectedness and importance of social medicine as well.

Thank you very much for your time and consideration. Please let me know if you have any questions about this initiative.

Kind regards,

[your name]

[END OF TEMPLATE]

------CLINICAL OPPORTUNITIES-------

Good morning (afternoon)

My name is _______, a medical student at the University of Ottawa.

I am part of a team of medical students that are putting together a Social Medicine Network - an online resource of social or community health services offering community contacts and clinical opportunities. This is a student-run project to create a website for other students to be able to visit and find learning opportunities in various topics related to social accountability. We would be honoured to list your [name/service/organization/initiative].

I am writing to you because we are interested in listing (contact) as a resource in the Network as a clinical observership, shadowing, or elective opportunity. This would make this opportunity (or contact), along with others, more visible to students who are interested in this work and/or learning more. This service can help connect students with clinicians and other healthcare providers for clinical opportunities.

Please let me know if (contact x or service x) is interested in being included in this initiative. If so, we will ask for some basic information in order to complete the listing on the Network.

Ultimately, we hope the Social Medicine Network will help bridge the gap between social and clinical medicine, allowing students to appreciate the interconnectedness and importance of social medicine as well.

Thank you very much for your time and consideration. Please let me know if you have any questions about this initiative.

Kind regards,

[your name]

--OR, if no clear volunteer opportunity exists…--

I am writing to you because I am wondering if (x service) offers any learning opportunities, volunteer placements, or observerships that could be included in the Network. This would allow medical students to become more aware of learning opportunities offered at (x service).

Please let me know if (service x) are interested in being included in this initiative. If so, we will ask for some basic information in order to complete the listing on the Network.

Thank you very much for your consideration. Please let me know if you have any questions about this initiative.

Sincerely,

X
